# Postoperative hypophosphatemia as a predictor of complications after pancreaticoduodenectomy: Insights from a tertiary gastrointestinal surgery center

**DOI:** 10.1097/MD.0000000000043660

**Published:** 2025-08-01

**Authors:** Erol Aksoy, Alper Güven, Muhammet Kadri Çolakoğlu, Osman Aydin, Yiğit Mehmet Özgün, Zeynep Göktaş, Erdal Birol Bostanci

**Affiliations:** a Department of Gastrointestinal Surgery, Health Sciences University Ankara Bilkent City Hospital, Ankara, Turkey; b Department of Nutrition and Dietetics, Hacettepe University, Ankara, Turkey.

**Keywords:** hypophosphatemia, pancreaticoduodenectomy, pancreaticojejunostomy leakage, postoperative complications, serum phosphate levels

## Abstract

Hypophosphatemia is a common metabolic disturbance following major abdominal surgeries, including pancreaticoduodenectomy (PD), and has been associated with an increased risk of postoperative complications. This study aims to investigate the association of serum phosphate levels with postoperative complications in patients undergoing PD at a tertiary gastroenterological surgery center. This retrospective study included 190 patients who underwent PD between February 2019 and December 2022. Serum phosphate levels were measured perioperatively. Hypophosphatemia was defined as serum phosphate levels < 2.5 mg/dL. Postoperative complications, including pancreaticojejunostomy (PJ) leakage, wound infection, and delayed gastric emptying, were recorded. Statistical analyses were performed to assess the correlation between hypophosphatemia and postoperative complications. Hypophosphatemia was observed in 148 patients (77.89%), with the lowest phosphate levels recorded on the 3rd postoperative day (2.17 ± 0.05 mg/dL, *P* < .05). Complications developed in 126 patients (66.3%). Hypophosphatemia was significantly more common in patients with complications (82.5% vs 68.8%, *P* = .03). PJ leakage was observed in 58 patients (30.5%), with significantly lower serum phosphate levels on the 3rd and 5th postoperative days (*P* = .003, *P* = .005). Overall, hypophosphatemia was prevalent after PD and associated with increased rates of complications, particularly with PJ leakage. Hypophosphatemia is associated with an increased risk of postoperative complications after PD, particularly with PJ leakage. Early identification and management of low phosphate levels in these patients could improve clinical outcomes and patient recovery.

## 1. Introduction

Phosphate is essential for numerous vital physiological functions, including adenosine triphosphate (ATP) production, mitochondrial activity, pH buffering, signal transduction, glycolysis, and the synthesis of 2,3-diphosphoglycerate (2,3-DPG). It also plays a critical role in membrane transport, serving as a structural component of deoxyribonucleic acid (DNA) and ribonucleic acid (RNA). Phosphate homeostasis is regulated by several organs, including the bones, parathyroid glands, liver, kidneys, and small intestine.^[1,2]^ However, in acute illness or the postoperative period, these regulatory mechanisms may become insufficient, often leading to hypophosphatemia in critically ill patients. Disruptions in phosphate balance have been associated with serious complications, such as arrhythmias, respiratory muscle weakness, red blood cell dysfunction, coma, and seizures.^[3]^

Hypophosphatemia is observed in approximately 44.8% of patients in general surgical intensive care units, with its prevalence increasing up to 67% after major surgical procedures.^[4]^ Hypophosphatemia has been frequently reported after many abdominal surgery procedures, such as hepatic, pancreatic, gastric and colorectal operations. In a study involving 8034 patients who underwent pancreatic, colorectal and gastric surgery, hypophosphatemia was observed in the majority of patients and associated with organ-specific complications.^[5]^

Pancreaticoduodenectomy is a complex surgical procedure associated with substantial morbidity (up to 70%) and mortality rates (up to 30%), despite advancements in surgical techniques and postoperative care.^[6]^ Major complications of PD include pancreatic fistula, hemorrhage, delayed gastric emptying, surgical site infections, chyle leaks, and pancreatic insufficiency.^[7]^ Hypophosphatemia is a common postoperative finding in patients undergoing PD. Mueller et al reported that patients who developed postoperative pancreatic fistulas (POPF) exhibited significantly and persistently lower phosphate levels.^[8]^ Similarly, Wong et al reported that 85% of their patients experienced some degree of hypophosphatemia during their hospitalization. They also found that distal pancreatectomy was more frequently associated with postoperative hypophosphatemia, and that patients with severe hypophosphatemia had a significantly higher risk of fistula-related complications.^[9]^

The clinical significance of hypophosphatemia lies in its potential role as an early indicator of postoperative complications, particularly in high-risk surgeries such as PD. Hypophosphatemia is associated with impaired cellular metabolism, immune dysfunction, and delayed recovery, all of which can contribute to increased postoperative morbidity. Early detection and management of hypophosphatemia may help reduce the risk of complications, including pancreatic fistulas, infections, and delayed wound healing. While hypophosphatemia has been reported after various abdominal surgeries, limited data are available regarding its timing, severity, and specific association with complications, such as PJ leak in patients undergoing PD. This study aims to fill this gap by systematically evaluating postoperative serum phosphate trends, categorizing the degree of hypophosphatemia, and investigating their relationship with specific postoperative complications – particularly with pancreaticojejunostomy leakage – in a large cohort of patients who underwent PD at a tertiary gastrointestinal surgery center.

## 2. Materials and methods

### 2.1. Study design and setting

This retrospective study was conducted at the Department of Gastrointestinal Surgery, Ankara Bilkent City Hospital, a high-volume tertiary referral center in Ankara, Türkiye. The study included patients who underwent pancreaticoduodenectomy between February 2019 and December 2022. Institutional ethics committee approval was obtained prior to data collection (Approval No: E2-22-2970).

### 2.2. Patient selection

All patients who underwent PD during the study period were screened for inclusion. Patients were excluded if they were under 18 years of age, lacked postoperative serum phosphate measurements, or had a preoperative estimated glomerular filtration rate below 60 mL/min/1.73 m².

### 2.3. Data collection

Demographic and clinical data were retrospectively retrieved from hospital electronic records. Variables included age, sex, comorbidities (diabetes mellitus, cardiovascular disease, hypertension), America Society of Anesthesiologists (ASA) classification, underlying disease, tumor stage, operative details, postoperative complications, and mortality within 30 days. The type of pancreatic anastomosis and use of intraoperative pancreatic stents were also recorded.

### 2.4. Definitions of outcomes

Postoperative complications were recorded and classified, with a specific focus on pancreaticojejunostomy leakage, wound infections, intra-abdominal collections, pneumonia, pleural effusion, and delayed gastric emptying. PJ leakage was defined according to the International Study Group on Pancreatic Fistula (ISGPF) criteria as a drain amylase concentration more than 3 times the upper limit of normal serum amylase on postoperative day 3 or later. PJ leaks were categorized as biochemical (grade A) or clinically relevant (grade B or C).

### 2.5. Phosphate monitoring protocol

Serum phosphate levels were measured within 1 week preoperatively and daily postoperatively until discharge. Hypophosphatemia was defined as a serum phosphate concentration <2.5 mg/dL, based on established clinical thresholds.^[9–11]^ The degree of hypophosphatemia was further categorized as severe (<2.0 mg/dL), moderate (2.0–2.5 mg/dL), or normal (>2.5 mg/dL).

### 2.6. Statistical analyses

Statistical analyses were performed using SPSS 22.0 (IBM, USA). Descriptive statistics were presented as mean ± standard error, median and range, or frequency and percentage, depending on the variable type and distribution. The Kolmogorov–Simonov test was used to test the distribution of variables. Independent sample t test and paired sample t test were used for comparison of 2 group means. One-way ANOVA was used for comparison of more than 2 group means, with Levene test ensuring variance homogeneity. post hoc analyses was performed using Bonferroni for pairwise comparisons and adjusting for multiple comparisons. Repeated measures ANOVA was used to analyze changes in continuous variables across multiple time points. Categorical relationships between variables were explored using a chi-square test of independence. Cochran Q test was used for nonparametric analysis of categorical variables across repeated measures. Multivariate logistic regression analysis was performed to identify independent predictors of PJ leakage. Variables with clinical relevance and/or *P* < .1 in univariate analysis were included in the model. Odds ratios (ORs) with 95% confidence intervals (CIs) were reported. A *P*-value of < .05 was considered statistically significant.

## 3. Results

### 3.1. Patient demographics and clinical characteristics

A total of 190 patients were included in the study. Demographic and clinical characteristics are listed in Table 1. The mean age was 60.7 ± 0.78 years, and the majority of patients were male (63.7%). According to the ASA classification, 5 patients were ASA ≥ 3. Comorbidities included diabetes mellitus in 81 patients, cardiovascular disease in 42 patients, and hypertension in 88 patients. Ten patients (5.2%) died within a month postoperatively. The mean hospital stay was 20 days. Common bile duct stents were present in 94 patients (49.5%). Blumgart anastomosis was performed in 22 (11.6%) patients, pancreaticogastrostomy in 1 (0.5%) patient, pancreaticojejunostomy in 2 (1%) patients and wirsungojejunostomy in 165 (86.8%) patients. Intraoperative internal pancreatic stents were placed in 133 patients (70%). The mean diameter of the Wirsung duct was 4.67 ± 0.16 mm. Pancreas consistency was soft in 124 patients (65.3%) and firm in 66 patients (34.7%). Histopathological diagnoses included benign lesions in 10 patients, periampullary tumors in 156 patients, intraductal papillary mucinous neoplasm (IPMN) in 10 patients, and neuroendocrine tumors (NET) in 14 patients. Tumor stage and diameter are presented in Table 1.

**Table 1:** Demographic and clinical characteristics of patients.

	n = 190
Age	60.7 ± 0.7
Gender	
Female	69 (32.3%)
Male	121 (63.7%)
ASA ≥ 3	5 (2.6%)
Diabetes mellitus	81 (42.6%)
Cardiac comorbidities	41 (21.6%)
Hypertension	88 (46.3%)
Mortality	10 (5.3%)
Hospital stay (day)	20 ± 13.3
Preoperative common bile duct stents	94 (49.5%)
Pancreas anastomosis	
Blumgart	22 (11.6)
PG	1 (0.5%)
PJ	2 (1%)
WJ	165 (86.8%)
Intraoperative pancreatic stent	133 (70%)
Wirsung size (mm)	4.67 ± 0.16
Pancreas texture	
Soft	124 (65.3%)
Firm	66 (34.7%)
Pathological diagnosis	
Benign	10 (5.3%)
Periampullary tumor	156 (82.1%)
IPMN	10 (5.3%)
Neuroendocrine tumor	14 (7.3%)
Tumor size (cm)	3.13 ± 1.57
Stage	
Stage 1	22 (11.6%)
Stage 2	61 (32.1%)
Stage 3	80 (42.1%)

ASA = America Society of Anesthesiologists, IPMN = intraductal papillary mucinous neoplasm, PG = pancreaticogastrostomy, PJ = pancreaticojejunostomy, WJ = wirsungojejunostomy.

### 3.2. Postoperative complications

Major and minor postoperative complications were observed in 126 patients (66.3%) who underwent PD. PJ leakage occurred in 58 patients (30.5%). Among these, 27 patients (46.5%) were classified as having a biochemical leak, 30 patients (51.7%) grade B fistulas, and 1 patient (1.7%) grade C fistula. The other type of complications comprised wound infection in 97 patients (51.1%), intra-abdominal fluid collection in 47 patients (24.7%), pneumonia in 20 patients (10.5%), pleural effusion in 10 patients (5.3%) and delayed gastric emptying in 9 patients (4.7%) (Table 2).

**Table 2:** Pancreaticoduodenectomy complications.

	n = 190
Total complications	126 (66.3%)
PJ leakage	58 (30.5%)
Grade a	27 (14.2)
Grade b	30 (15.8%)
Grade c	1 (0.5%)
Others	
Wound infection	97 (51.1%)
Intra-abdominal collection	47 (24.7%)
Pneumonia	20 (10.5%)
Pleural effusion	10 (5.3%)
Delayed gastric emptying	9 (4.7%)
Hepaticojejunostomy leakage	4 (2.1%)
Chylous acid	4 (2.1%)
Pulmonary embolism	3 (1.6%)
Acute kidney failure	3 (1.6%)
Wound dehiscence	3 (1.6%)
Gastrointestinal bleeding	2 (1%)
Pseudoaneurysm	2 (1%)
Intra-abdominal bleeding	1 (0.5%)
Diarrhea	1 (0.5%)
Enterocutaneous fistula	1 (0.5%)
Acute cerebrovascular occlusion	1 (0.5%)

PJ = pancreaticojejunostomy.

### 3.3. Postoperative serum phosphate levels

The preoperative mean serum phosphate level of all patients was 3.61 ± 0.04 mg/dL. Postoperative mean serum phosphate levels declined significantly: 2.63 ± 0.06 mg/dL on postoperative day (POD) 1, 2.17 ± 0.05 mg/dL on POD 3, and 2.70 ± 0.05 mg/dL on POD 5. The lowest mean phosphate level was observed on POD 3 (*P* < .05). Overall, postoperative hypophosphatemia was present in 148 of 190 patients (77.89%). Specifically, hypophosphatemia was detected in 89 patients (46.84%) on POD 1, in 132 patients (69.47%) on POD 3, and in 78 patients (41.05%) on POD 5, with the highest incidence occurring on POD 3 (*P* < .05). Among patients who developed hypophosphatemia, the mean serum phosphate levels were 2.02 ± 0.05 mg/dL on POD 1, 1.83 ± 0.06 mg/dL on POD 3, and 2.06 ± 0.05 mg/dL on POD 5. The serum phosphate level was the lowest on the 3rd postoperative day (*P* < .05).

Postoperative serum phosphate levels were categorized into 3 groups: <2.0 mg/dL (severe hypophosphatemia), 2.0 to 2.5 mg/dL (moderate hypophosphatemia), and > 2.5 mg/dL (normal). According to this classification, on POD 1, 45 patients (23.7%) had severe hypophosphatemia, 55 (28.9%) had moderate hypophosphatemia, and 90 patients (47.4%) had normal phosphate levels. On the POD 3, 94 patients (49.5%) had severe, 42 (22.1%) moderate, and 54 (28.4%) normal phosphate levels. On the POD 5, 29 patients (15.3%) had severe, 57 (30.3%) moderate, and 104 (54.7%) normal phosphate levels. Severe hypophosphatemia peaked on the POD 3, affecting nearly half of the patients (49.5%) (*P* < .05).

### 3.4. Association between hypophosphatemia and postoperative complications

Postoperative morbidity occurred in 126 patients. Among these, hypophosphatemia was present in 104 patients (82.5%) who developed complications, while 44 patients with hypophosphatemia did not experience complications (*P* = .03). Subgroup analysis revealed no significant association between hypophosphatemia and complications on POD 1; however, a significant correlation was observed on POD 3 and 5 (*P* = .013, OR = 2.236, 95% CI: 1.18–4.24 and *P* = .001, OR = 3.200, 95% CI: 1.62–6.29, respectively) (Table 3). Mean serum phosphate levels on POD 3 and 5 were significantly lower in the complication group compared to those without complications (2.09 ± 0.05 vs 2.33 ± 0.09 mg/dL, *P* = .016 and 2.59 ± 0.06 vs 2.91 ± 0.09 mg/dL, *P* = .003, respectively) (Table 4). When stratified by phosphate levels (<2.0 mg/dL [severe hypophosphatemia], 2.0–2.5 mg/dL, >2.5 mg/dL), complication rates were significantly higher in patients with severe hypophosphatemia on the POD 3, whereas patients with phosphate levels > 2.5 mg/dL had significantly lower complication rates on both POD 3 and 5 (Table 5).

**Table 3:** Patients with postoperative hypophosphatemia and presence of complications.

	Present (n = 126)	Non-present (n = 64)	Total (n)	*P*	OR	95% CI
Hypophosphatemia*	104 (82.5%)	44 (68.8%)	148	***.03***	2.149	1.07–4.33
1st day post-op	62 (49.2%)	27 (42.2%)	89	.35	1.328	0.72–2.43
3rd day post-op	95 (75.4%)	37 (57.8%)	132	***.013***	2.23	1.18–4.24
5th day post-op	63 (50.0%)	15 (23.8%)	78	***.001***	3.20	1.62–6.29

The bold–italic values indicate statistically significant results (*P* < .05).

* Hypophosphatemia: number of patients.

**Table 4:** Pre- and postoperative mean serum phosphate levels versus the presence of complications.

	Present (n = 126)	Non-present (n = 64)	Total (n = 190)	*P*
Serum phosphate levels (mg/dL)				
Pre-op	3.62 ± 0.06	3.59 ± 0.08	3.61 ± 0.04	.742
1st day post-op	2.67 ± 0.08	2.57 ± 0.08	2.63 ± 0.06	.438
3rd day post-op	2.09 ± 0.05	2.33 ± 0.09	2.17 ± 0.05	***.016***
5th day post-op	2.59 ± 0.06	2.91 ± 0.09	2.70 ± 0.05	***.003***

The bold–italic values indicate statistically significant results (*P* < .05).

**Table 5:** Postoperative range of serum phosphate levels and presence of complications

	Present (n = 126)	Non-present (n = 64)	Total (n = 190)
Serum phosphate (mg/dL)			
Pre-op			
<2.00	1 (0.8%)	–	1 (0.5%)
2.00–2.50	6 (4.8%)	4 (6.2%)	10 (5.3%)
>2.50	119 (94.4%)	60 (93.8%)	179 (94.2%)
1st day post-op			
<2.00	32 (25.4%)	13 (20.3%)	45 (23.7%)
2.00–2.50	34 (27.0%)	21 (32.8%)	55 (28.9%)
>2.50	60 (47.6%)	30 (46.9%)	90 (47.4%)
3rd day post-op			
<2.00	74 (58.7%)	20 (31.3%)	94 (49.5%)
2.00–2.50	24 (19.1%)	18 (28.1%)	42 (22.1%)
>2.50	28 (22.2%)	26 (40.6%)	54 (28.4%)
5th day post-op			
<2.00	21 (16.7%)	8 (12.5%)	29 (15.3%)
2.00–2.50	46 (36.5%)	11 (17.2%)	57 (30.0%)
>2.50	59 (46.8%)	45 (70.3%)	104 (54.7%)

Marginal homogeneity test, *P* > .001 = Serum phosphate level distributions were significantly different between each repeated tests in both groups, except general complication non-present group’s 1st day post-op and 3rd day post-op measurements.

### 3.5. Hypophosphatemia and pancreaticojejunostomy leakage

PJ leak was observed in 58 patients. Among those with PJ leakage, 50 patients (86.2%) had hypophosphatemia compared to 98 patients (74.2%) without PJ leakage (*P* = .067). A significant correlation between hypophosphatemia and PJ leakage was found on the POD 3 and 5 (day 3: *P* = .003, OR = 3.214, 95% CI: 1.15–7.11; day 5: *P* = .004, OR = 2.523, 95% CI: 1.34–4.75) (Table 6).

**Table 6:** Patients with post-op hypophosptatemia and presence of pancreticojejunostomy leak.

	Present (n = 58)	Non-present (n = 132)	Total (n)	*P*
Hypophosphatemia*	50 (86.2%)	98 (74.2%)	148	.06
1st day post-op	28 (48.3%)	61 (46.2%)	89	.79
3rd day post-op	49 (84.5%)	83 (62.9%)	132	***.003***
5th day post-op	33 (56.9%)	45 (34.4%)	78	***.004***

The bold–italic values indicate statistically significant results (*P* < .05).

* Hypophosphatemia: number of patients.

Biochemical PJ leak was observed in 27 patients (14.2%), while grade B PJ leak occurred in 30 patients (15.8%). A significant difference was found between these groups regarding the incidence of hypophosphatemia on the POD 3 (*P* = .029, OR = 9.043, 95% CI: 1.05–77.8). Additionally, hypophosphatemia detected on the POD 5 was significantly associated with both wound infection (*P* = .003, OR = 2.432, 95% CI: 1.34–4.41) and intra-abdominal infection (*P* = .004, OR = 2.712, 95% CI: 1.38–5.33).

Patients who developed PJ leak had significantly lower serum phosphate levels on the POD 3 (1.97 ± 0.07 mg/dL vs 2.27 ± 0.06 mg/dL, *P* = .003) and on the POD 5 (2.49 ± 0.07 mg/dL vs 2.80 ± 0.06 mg/dL, *P* = .005). There was no significant difference in serum phosphate levels between patients with and without PJ leakage on the preoperative day and the POD 1 (Table 7). Similarly, patients who experienced complications other than PJ leak also showed significantly lower serum phosphate levels on the POD 3 (2.09 ± 0.05 mg/dL vs 2.33 ± 0.09 mg/dL, *P* = .016) and the POD 5 (2.59 ± 0.06 mg/dL vs 2.91 ± 0.09 mg/dL, *P* = .003).

**Table 7:** Pre- and post-op mean serum phosphate levels by the presence of pancreaticojejunostomy leak.

	Present (n = 58)	Non-present (n = 132)	Total (n = 190)	*P*
Serum phosphate levels (mg/dL)				
Pre-op	3.65 ± 0.09	3.59 ± 0.05	3.61 ± 0.04	.680
1st day post-op	2.60 ± 0.11	2.65 ± 0.07	2.63 ± 0.06	.507
3rd day post-op	1.97 ± 0.07	2.27 ± 0.06	2.17 ± 0.05	***.003***
5th day post-op	2.49 ± 0.07	2.80 ± 0.06	2.70 ± 0.05	***.005***

The bold–italic values indicate statistically significant results (*P* < .05).

The incidence of PJ leak was significantly higher in patients with serum phosphate levels below 2.00 mg/dL on the POD 3, while PJ leak rates significantly decreased in patients with phosphate levels above 2.50 mg/dL on both the POD 3 and 5 (Table 8).

**Table 8:** Post-op range of serum phosphate levels and pancreaticojejunostomy leak.

	Present (n = 58)	Non-present (n = 132)	Total (n = 190)
Serum phosphate (mg/dL)			
Pre-op			
<2.00	–	1 (0.8%)	1 (0.5%)
2.00–2.50	5 (8.6%)	5 (3.8%)	10 (5.3%)
>2.50	53 (91.4%)	126 (95.5%)	179 (94.2%)
1st day post-op			
<2.00	16 (27.6%)	29 (22.0%)	45 (23.7%)
2.00–2.50	15 (25.9%)	40 (30.3%)	55 (28.9%)
>2.50	27 (46.6%)	63 (47.7%)	90 (47.4%)
3rd day post-op			
<2.00	41 (70.7%)	53 (40.2%)	94 (49.5%)
2.00–2.50	8 (13.8%)	34 (25.8%)	42 (22.1%)
>2.50	9 (15.5%)	45 (34.0%)	54 (28.4%)
5th day post-op			
<2.00	10 (17.2%)	19 (14.4%)	29 (15.3%)
2.00–2.50	26 (44.8%)	31 (23.5%)	57 (30.0%)
>2.50	22 (37.9%)	82 (62.1%)	104 (54.7%)

Marginal homogeneity test, *P* > .001 = Serum phosphate level distributions were significantly different between each repeated tests in both groups.

In the multivariate logistic regression analysis, hypophosphatemia on postoperative day 3 was independently associated with an increased risk of PJ leakage (OR: 2.14; 95% CI: 1.08–4.23; *P* = .029). Other variables, including age (OR: 1.02; 95% CI: 0.98–1.07; *P* = .210), gender (OR: 1.31; 95% CI: 0.59–2.89; *P* = .503), presence of comorbidities (OR: 1.27; 95% CI: 0.52–3.09; *P* = .592), pancreatic texture (OR: 1.68; 95% CI: 0.91–3.10; *P* = .094), and type of pancreatic anastomosis (OR: 0.81; 95% CI: 0.47–1.06; *P* = .180), were not statistically significant predictors of PJ leakage (Table 9).

**Table 9:** Multivariate logistic regression analysis for predictors of pancreaticojejunostomy leakage.

Variable	OR	95% CI	*P*
Hypophosphatemia on 3rd day post-op	2.14	1.08–4.23	**.029**
Age	1.02	0.98–1.07	.210
Gender	1.31	0.59–2.89	.503
Comorbidities	1.27	0.52–3.09	.592
Pancreas texture	1.68	0.91–3.10	.094
Type of pancreas anastomosis	0.81	0.47–1.06	.180

The bold value indicates statistically significant results (*P* < .05).

## 4. Discussion

Hypophosphatemia is a common metabolic abnormality following major abdominal surgeries, including PD, and may be associated with postoperative complications such as fistula formation. Therefore, it may possibly be an early marker to detect fistulas and other complications after pancreatectomy. Despite its high prevalence in the postoperative period, its correlation with complications, particularly related to pancreatic surgeries, has not been investigated in the literature. This study addresses this gap by analyzing the relationship between serum phosphate levels and postoperative outcomes in patients undergoing PD. Our results demonstrate a significant association between hypophosphatemia and increased rates of postoperative complications, including PJ leakage, suggesting a potential value of phosphate monitoring in the postoperative management of these patients.

As mentioned before, hypophosphatemia is seen in the majority of patients undergoing major abdominal surgeries. Several studies have previously reported the occurrence of postoperative hypophosphatemia after major gastrointestinal surgeries; however, only a few have specifically focused on chronology and clinical implications following pancreaticoduodenectomy. Our findings align with those of Mueller et al, who reported that persistently low phosphate levels were associated with postoperative pancreatic fistula formation following pancreatectomy.^[8]^ Similarly, Wong et al reported that hypophosphatemia was common after pancreatic surgery, with a correlation between its severity and fistula-related complications.^[9]^ By stratifying phosphate levels across specific postoperative days, our study adds a temporal resolution to this association and identifies the third postoperative day as the critical time when severe hypophosphatemia is most prevalent and most strongly associated with complications.

The biological mechanisms linking hypophosphatemia to poor surgical outcomes are multifactorial. Phosphate is essential for ATP synthesis, which is vital for cellular repair, immune function, and energy metabolism. During the postoperative catabolic state, phosphate shifts intracellularly due to insulin-mediated cellular uptake, increased metabolic demand, and inadequate nutritional intake. This state of phosphate depletion can impair leukocyte function, reduce diaphragmatic contractility, and compromise tissue oxygenation, all of which may contribute to impaired wound healing and increased susceptibility to infection and anastomotic leakage.^[5,12]^ These mechanisms are particularly relevant in PD, a high-risk operation with anastomoses that requires a delicate balance between healing and breakdown.

The observed clinical association suggests that early postoperative serum phosphate levels may serve as a surrogate biomarker for patients at risk of developing PJ leakage or other complications. This could have meaningful implications to be included in perioperative monitoring protocols. For instance, serial phosphate measurements – particularly on postoperative days 1 through 5 – could be incorporated into standard postoperative care. More importantly, patients with severe hypophosphatemia could be identified for targeted nutritional interventions or closer surveillance. While causality cannot be inferred from our observational design, the strength and consistency of the associations underscore the potential clinical utility of phosphate as a risk stratification tool.

Despite the observed associations, several potential confounders must be acknowledged. Nutritional status, which is often suboptimal in patients with pancreatic malignancies, can influence both phosphate levels and wound healing capacity. Similarly, comorbidities such as diabetes mellitus or chronic renal insufficiency, although excluded if severe in this cohort, may still contribute to altered phosphate metabolism and immune responses. Variability in surgical technique, pancreatic texture, or pancreatic duct diameter could also modulate risk independently of phosphate levels.

Pancreaticojejunostomy leakage is one of the most challenging complications after pancreaticoduodenectomy, associated with prolonged hospital stay, high morbidity and mortality. Our study demonstrated that hypophosphatemia was significantly associated with higher rates of PJ leakage and overall postoperative complications. PJ leakage, a critical and challenging complication after PD, was more common in patients with severe hypophosphatemia (≤2.0 mg/dL). This finding is consistent with the hypothesis that low phosphate levels impair wound healing, reduce cellular energy availability, and compromise immune function, thereby predisposing patients to anastomotic dehiscence and infection.

Previous studies have reported the prevalence of hypophosphatemia in major abdominal surgeries, but only a few have focused on PD. For instance, Sadot et al found a significant association between low phosphate levels and organ-specific complications, including anastomotic leaks, following pancreatic, colorectal, and gastric resections.^[5]^ Similarly, Grikyte and Ignatavicius highlighted the delayed recovery of hypophosphatemia in patients with leak-related complications after gastrointestinal surgeries.^[13]^ Our findings contribute to this knowledge by suggesting a predictive value of hypophosphatemia for PJ leakage and overall complications after PD. Moreover, we observed that phosphate levels began to recover by the fifth postoperative day with appropriate nutritional and phosphate supplementation, underscoring the reversible nature of this metabolic disturbance when addressed promptly.

The pathophysiological link between hypophosphatemia and surgical complications is multifactorial. In our study, patients with severe hypophosphatemia had significantly lower phosphate levels on postoperative days 3 and 5, correlating with higher rates of PJ leakage and infections, including wound and intra-abdominal infections. These findings suggest that hypophosphatemia may serve as an early marker for identifying high-risk patients who require intensified monitoring and intervention.

The high prevalence of hypophosphatemia observed in this study (77.9%) highlights the need for routine postoperative monitoring of serum phosphate levels in patients undergoing PD. An increase in phosphorus levels is detected after the 3rd day with oral, enteral and/or parenteral nutritional support and phosphorus supplementation in the postoperative period. Early identification of hypophosphatemia allows for timely nutritional and pharmacological interventions, potentially reducing the risk of complications. For instance, targeted phosphate supplementation, combined with nutritional support, could mitigate the adverse effects of hypophosphatemia on wound healing and immune function. Future studies should explore the efficacy of standardized protocols for managing hypophosphatemia in surgical patients.

This study has several strengths, including its focus on a specific and high-risk surgical population and the comprehensive analysis of postoperative serum phosphate levels. However, it also has limitations. The retrospective design may introduce selection bias, and the single-center setting limits the generalizability of our findings. potential measurement bias due to timing variability in phosphate testing, A lack of detailed data on phosphate supplementation timing and dose were the other limitations. Additionally, we did not assess the long-term outcomes of patients with hypophosphatemia, which could provide further insights into its clinical impact. Given the significant association between hypophosphatemia and postoperative complications, future research should focus on prospective, multicenter studies to validate our findings. Investigating the cost-effectiveness of routine phosphate monitoring and supplementation in improving postoperative outcomes is also warranted. Furthermore, exploring the role of hypophosphatemia in other surgical populations could broaden our understanding of its clinical relevance.

## 5. Conclusion

Hypophosphatemia is a common and clinically significant metabolic disturbance following PD, strongly associated with increased rates of PJ leakage and overall complications. Routine monitoring and early intervention for hypophosphatemia may enhance patient outcomes, reduce morbidity, and improve recovery. This study underscores the importance of integrating serum phosphate monitoring into postoperative care protocols in patients undergoing complex abdominal surgeries.

## Author contributions

**Conceptualization:** Erol Aksoy, Muhammet Kadri Çolakoğlu.

**Data curation:** Erol Aksoy, Muhammet Kadri Çolakoğlu, Osman Aydin, Zeynep Göktaş.

**Formal analysis:** Osman Aydin, Zeynep Göktaş.

**Investigation:** Erol Aksoy, Muhammet Kadri Çolakoğlu, Zeynep Göktaş.

**Methodology:** Erol Aksoy, Muhammet Kadri Çolakoğlu, Zeynep Göktaş.

**Project administration:** Erol Aksoy, Muhammet Kadri Çolakoğlu.

**Resources:** Erol Aksoy, Alper Güven, Osman Aydin.

**Software:** Alper Güven.

**Supervision:** Yiğit Mehmet Özgün, Erdal Birol Bostanci.

**Validation:** Osman Aydin, Yiğit Mehmet Özgün, Erdal Birol Bostanci.

**Visualization:** Yiğit Mehmet Özgün, Erdal Birol Bostanci.

**Writing – original draft:** Erol Aksoy, Yiğit Mehmet Özgün, Erdal Birol Bostanci.

**Writing – review & editing:** Muhammet Kadri Çolakoğlu.
